# A step forward for Shiga toxin-producing *Escherichia coli* identification and characterization in raw milk using long-read metagenomics

**DOI:** 10.1099/mgen.0.000911

**Published:** 2022-11-24

**Authors:** Sandra Jaudou, Carlus Deneke, Mai-Lan Tran, Elisabeth Schuh, André Goehler, Fabien Vorimore, Burkhard Malorny, Patrick Fach, Josephine Grützke, Sabine Delannoy

**Affiliations:** ^1^​ COLiPATH Unit, Laboratory for Food Safety, ANSES, Maisons-Alfort, France; ^2^​ National Study Center for Sequencing, Department of Biological Safety, German Federal Institute for Risk Assessment, Berlin, Germany; ^3^​ Genomics Platform IdentyPath, Laboratory for Food Safety, ANSES, Maisons-Alfort, France; ^4^​ National Reference Laboratory for *Escherichia coli* including VTEC, Department of Biological Safety, German Federal Institute for Risk Assessment, Berlin, Germany

**Keywords:** genome assembly, isolation-independent identification, long-read sequencing, metagenomics, raw milk, Shiga toxin-producing *Escherichia coli *(STEC)

## Abstract

Shiga toxin-producing *Escherichia coli* (STEC) are a cause of severe human illness and are frequently associated with haemolytic uraemic syndrome (HUS) in children. It remains difficult to identify virulence factors for STEC that absolutely predict the potential to cause human disease. In addition to the Shiga-toxin (*stx* genes), many additional factors have been reported, such as intimin (*eae* gene), which is clearly an aggravating factor for developing HUS. Current STEC detection methods classically rely on real-time PCR (qPCR) to detect the presence of the key virulence markers (*stx* and *eae*). Although qPCR gives an insight into the presence of these virulence markers, it is not appropriate for confirming their presence in the same strain. Therefore, isolation steps are necessary to confirm STEC viability and characterize STEC genomes. While STEC isolation is laborious and time-consuming, metagenomics has the potential to accelerate the STEC characterization process in an isolation-free manner. Recently, short-read sequencing metagenomics have been applied for this purpose, but assembly quality and contiguity suffer from the high proportion of mobile genetic elements occurring in STEC strains. To circumvent this problem, we used long-read sequencing metagenomics for identifying *eae*-positive STEC strains using raw cow's milk as a causative matrix for STEC food-borne outbreaks. By comparing enrichment conditions, optimizing library preparation for MinION sequencing and generating an easy-to-use STEC characterization pipeline, the direct identification of an *eae*-positive STEC strain was successful after enrichment of artificially contaminated raw cow's milk samples at a contamination level as low as 5 c.f.u. ml^−1^. Our newly developed method combines optimized enrichment conditions of STEC in raw milk in combination with a complete STEC analysis pipeline from long-read sequencing metagenomics data. This study shows the potential of the innovative methodology for characterizing STEC strains from complex matrices. Further developments will nonetheless be necessary for this method to be applied in STEC surveillance.

## Data Summary

All basecalled and demultiplexed fastq files were deposited in the National Center for Biotechnology Information (NCBI) Sequence Read Archive (SRA) under BioProject PRJNA835223. The code for the STECmetadetector pipeline is freely available from GitLab (https://gitlab.com/bfr_bioinformatics/STECmetadetector).

**Impact Statement**

Shiga toxin-producing *Escherichia coli* (STEC) are food-borne pathogens that may cause severe human illnesses such as haemolytic uraemic syndrome. Current detection methods rely on time-consuming and laborious isolation steps for STEC characterization. Because short-read sequencing is not sufficient to span repeated sequences widely present in STEC genomes, we assessed the potential of long-read sequencing to identify and characterize STEC strains in an isolation-independent way. Here, we artificially contaminated raw cow's milk and showed that the inoculated *eae*-positive STEC O26 strain was identifiable in raw milk enriched at 37 °C in acriflavine-supplemented buffered peptone water, from an STEC concentration of 5 c.f.u. (ml raw milk)^−1^. For ease of analysis, we developed an automated STEC characterization pipeline. We present a complete workflow to identify and characterize STEC directly from raw cow's milk using long-read metagenomics and an assembly-based method. This study demonstrates the ability of long-read metagenomics to efficiently characterize *E. coli* strains directly from a complex matrix.

## Introduction

Shiga toxin-producing *Escherichia coli* (STEC) are a subset of diarrheagenic *E. coli *, which are known to cause various symptoms ranging from watery diarrhoea to haemorrhagic colitis (HC) or haemolytic uraemic syndrome (HUS) [1, 2]. Their main virulence factor is located on bacteriophages, named *stx*-phages, which encode the Shiga toxin, and are integrated into the *E. coli* chromosome [3]. STEC pathogenicity assessment is challenged by their capacity to acquire mobile genetic elements (MGEs) carrying various virulence factors.

Although the main STEC virulence property is the capacity to produce the Shiga toxin (Stx), their ability to acquire MGEs encoding virulence factors may increase the risk of severe human symptoms. Some STEC frequently causing HUS additionally carry a pathogenicity island named locus of enterocyte effacement (LEE). One of the most notable protein encoded by the LEE is intimin (*eae* gene), which is responsible for bacterial adhesion to the intestinal cells [4]. Hybrid STEC strains harbouring virulence factors of other *E. coli* pathotypes and causing HUS are increasingly described. In 2011, an O104:H4 strain expressing aggregative factors from enteroaggregative *E. coli* (EAEC) along with an *stx* gene was responsible for a HUS outbreak in both Germany and France [5]. More recently, an O80:H2 cross-pathotype harbouring extra-intestinal pathogenic *E. coli* (ExPEC) virulence markers and an *stx* gene was described in France [6]. STEC O80 now represents the second main cause of HUS in Europe [7–9].

Though STEC are mostly found in cattle gut microbiota, contaminated food products are a major source of human infections, especially bovine meat and dairy products [9, 10]. In Europe, most severe human cases caused by food consumption are generally associated with *eae*-positive STEC strains of the top five O-groups: O26, O157, O103, O145 and O111 [9]. STEC strains in food and animal feed are screened by real-time PCR on DNA extracted from an enriched food portion [11]. However, PCR performed on food enrichment samples is not sufficient to ensure that *stx*, *eae* and the O-group associated genes belong to the same strain. Consequently, isolation of the STEC from all *stx*-positive samples is attempted for further characterization.

STEC isolation steps are laborious, time-consuming and frequently unsuccessful due, in part, to the lack of selective media. When isolation of STEC strains fails, no strain characterization can be done using conventional approaches. We were interested in exploring metagenomics to facilitate STEC strain characterization from food samples by providing crucial information on virulence factors and typing, in an isolation-independent way. However, the high content of MGEs containing repetitive sequences in STEC complicates their assembly using short-read sequencing, which can result in more than 200 contigs [12]. Recently, long-read sequencing was developed to resolve highly repetitive sequences [13]. Different long-read sequencing technologies exist, among which the most commonly used were commercialized by Pacific Biosciences (PacBio) and Oxford Nanopore Technology (ONT) [13, 14]. The MinION platform, released in 2014 by Oxford Nanopore Technology, known for its portability and its ability to generate very long reads, has been increasingly tested for food-borne pathogen detection [15–20].

Genomic studies using MinION sequencing were conducted on isolated STEC strains and the generated long-reads were proven to be sufficient to identify crucial MGEs responsible for STEC pathogenicity, including plasmids, phages, virulence genes and antimicrobial-resistance genes, which may be lost when assembling with short reads [21]. While whole-genome sequencing still requires a bacterial isolation step for characterizing pure isolates, metagenomics based on sequencing the total DNA from the specimen –including micro-organisms and the matrix DNA – may provide the means to identify and characterize STEC in an isolation-independent way. So far, major studies using long-read metagenomics for identifying STEC were performed on artificial mixtures or artificially contaminated food portions. In 2018, Peritz and colleagues demonstrated the potential of MinION for resolving STEC O-groups from artificial DNA mixtures of five STEC isolates [22]. Metagenomics analysis of artificially contaminated beef, spinach, pasteurized milk or wastewater using STEC strains has demonstrated the utility of MinION sequencing for STEC detection or identification and its limits [17–19, 23].

The objective of this study was to develop a long-read sequencing-based method to characterize STEC without the need to perform a strain isolation step. Here, we used an *eae*-positive O26 STEC strain, since they are frequently associated with the most severe human cases in Europe. We chose an assembly-based approach to detect *stx* and *eae* genes on the same contig, as well as the O-group and H-type associated genes. Because the *stx*-prophage may integrate into the genome at different sites, the *stx* and *eae* genes might be distantly located by up to 2.1 Mb [24]. Due to the high fragmentation of the draft genomes resulting from short-read sequencing methods, these are not appropriate. In contrast, the reads obtained from MinION sequencing that can be up to 4 Mb in length have the potential of resolving repeated sequences and thereby of improving assembly contiguity. Long-read assemblers try to span repetitive regions by using either an overlap-layout-consensus (OLC) or a graph-based approach; thus, closing gaps and generating long contigs that can be as long as the chromosome itself [25, 26].

So far, no studies have been conducted to identify and characterize *eae*-positive STEC strains in raw milk using long-read metagenomics. We used raw milk as an example of a typical food product that is known to cause severe STEC outbreaks [27]. Our method aimed to assemble the *eae*-positive STEC genome in bovine raw milk using long-read metagenomics. Here, we artificially contaminated STEC-negative raw cow's milk using an *eae*-positive STEC strain of serotype O26:H11 and optimized its growth conditions. To facilitate long-read metagenomics data analysis, we developed a complete pipeline for *eae*-positive STEC characterization. In this study, we present an entire and accessible workflow for STEC identification and characterization when detected in raw cow's milk using an assembly-based method from long-read metagenomics data. It shows that metagenomics approaches using long-read technologies are becoming an easy and time-efficient alternative to classical STEC characterization methods, and could be suitable to identify and eliminate hazards for the consumer at an early stage in the near future.

## Methods

### Selected strains

For artificial contamination of raw cow's milk, *eae*-positive STEC (*stx-*positive and *eae*-positive) strains from the ANSES (French Agency for Food, Environmental and Occupational Health and Safety) collection were used. Two different *eae*-positive STEC strains of serotype O26:H11 isolated from bovine raw milk were selected: 4712-O26 (*eae*, *stx1a*) and 6423-O26 (*eae*, *stx1a*) (Table 1). Each strain has been previously characterized using both Illumina and Oxford Nanopore Technology sequencing methods [26]. Sequencing data for 4712-O26 and 6423-O26 were deposited in GenBank under accession numbers SRR18191504, SRR18191503, SRR18191500 and SRR18191499, within BioProject PRJNA808207.

**Table 1.** Characteristics of the inoculated STEC O26 strains for artificial contamination of raw milk

Isolate	Serotype	ST	*stx* subtype	Stx-phage insertion site	*eae* subtype	*stx*/*eae* distance	Isolation year	Isolation source
4712-O26	O26:H11	21	1a	*wrbA*	Beta1	1.9 Mb	2014	Bovine raw milk
6423-O26	O26:H11	21	1a	*wrbA*	Beta1	1.9 Mb	2015	Bovine raw milk

### *In silico* determination of the minimum genome coverage required for *eae*-positive STEC strain identification

Oxford Nanopore Technology sequencing data generated by Jaudou and colleagues using the MinION instrument from pure culture of 10 *eae*-positive STEC strains (Table S1, available with the online version of this article) were selected depending on their N50 value (9087–22 759 bp) [26]. Data were sub-sampled to various genome coverages (3×, 5×, 10×, 15×, 20×, 25×, 30×, 35×, 40×, 50×, 60×, 70×) using the random sub-sampling tool rasusa v0.6.0 [28] (https://github.com/mbhall88/rasusa), and further assembled using the default mode of three different long-read assemblers: Flye v2.8.1-b1676 (https://github.com/fenderglass/Flye), Raven v1.2.2 and v1.7.0 (https://github.com/lbcb-sci/raven), and Canu v2.1.1 (https://github.com/marbl/canu) [29–31]. The quality of the resulting assemblies was assessed with quast v5.0.2 [32] (http://quast.sourceforge.net/). Virulence genes were detected using genial v1.0 (an abricate v0.8.7 [11] wrapper; https://github.com/p-barbet/GENIAL) and the VFDB (Virulence Factor Database) version 2020-05-29 (https://github.com/tseemann/abricate/tree/master/db/vfdb). Results from genial were used to determine whether both *stx* and *eae* genes were on the same contig. Only when *stx* and *eae* virulence genes were present on a same contig, the coverage for the assembly was considered sufficient for *eae*-positive STEC identification.

### Raw milk screening for *eae*-positive STEC

Five fresh raw milk samples were collected from dairy cows kept on the experimental farm of the German Federal Institute for Risk Assessment (BfR, Berlin, Germany) or bought from a French farm located in the surroundings of Paris, and stored at +4 °C for a maximum of 1 day until use. For enrichment, 1 ml of raw milk was diluted 1 : 10 in buffered peptone water (BPW; bioMérieux or Mast Group) and incubated overnight either at 37 or 41.5 °C with agitation. After 18 to 20 h of enrichment, DNA from each enriched milk sample was extracted using InstaGene^TM^ matrix (Bio-Rad), following the manufacturer’s instructions. Enriched cultures (1 ml) were centrifuged at a minimum of 10 000 r.p.m. for 3 min and the raw milk fat layer was detached using either a sterile cotton swab or a pipet tip. The pellets were washed twice with 1 ml PBS before DNA extraction. Real-time PCR was performed on each sample for the detection of *stx1*, *stx2*, *eae* and *wecA* or *cdgR,* as described in Methods (in the Real-time PCR analysis of enriched raw milk section). The genetic markers *wecA* or *cdgR* were used as generic *E. coli* gene targets. STEC-negative raw milk samples were further used for artificial contamination.

### Artificial contamination of STEC-negative raw milk and enrichment conditions

Strains used for artificial contamination of raw milk were revived from 20–30 % (v/v) glycerol stock on TSYe (Tryptone soy yeast extract agar) or TSA (tryptic soy agar) plates (bioMérieux or Mast Group) and incubated overnight at 37 °C. One colony was transferred to brain heart infusion (BHI) broth (bioMérieux or Mast Group) and incubated overnight at 37 °C with rotation. Approximate bacterial concentrations of pure overnight cultures and 1 : 10 serial dilutions were determined by optical density measurement at 600 nm (OD_600_). Actual spike-in levels were calculated by triplicate plate counting on TSYe or TSA plates. Raw milk (1 ml) was initially inoculated with respective 1 ml STEC O26 cultures of 10^3^ c.f.u. ml^−1^ (1.25–1.78×10^3^ c.f.u. ml^−1^), 10^2^ c.f.u. ml^−1^ (1.6–2.63×10^2^ c.f.u. ml^−1^) or 10^1^ c.f.u. ml^−1^ (1.33×10^1^ c.f.u. ml^−1^); aiming for estimated levels of 0.5×10^3^, 0.5×10^2^ and 0.5×10^1^ c.f.u. ml^−1^ of STEC O26 in the raw milk. Each artificially contaminated sample was inoculated with a single STEC O26 strain. Artificially contaminated raw milk (2 ml) was incubated in BPW (9 ml) with or without acriflavine supplementation (final acriflavine concentration of 12 mg l^−1^) with agitation [International Organization for Standardization/technical specification (ISO/TS) 13136 : 2012] [11] and incubated for 18 to 20 h either at 37 or 41.5 °C. A negative control (raw milk not artificially contaminated with STEC) was similarly processed. All experiments were performed in triplicate.

### DNA extraction from enriched raw milk for MinION sequencing

The enriched raw milk (1 ml) was centrifuged and the raw milk fat layer was removed before the pellet was washed with 1× PBS as described above for raw milk screening. DNA extraction and purification was performed using the MasterPure complete DNA and RNA extraction and purification kit (Lucigen), following the manufacturer’s instructions and including an RNase A treatment for 30 min (Qiagen; 100 mg ml^−1^) [26]. Genomic DNA was quantified using a Qubit 3.0 fluorometer and the broad range kit (Agilent), and quality control of the DNA extracts was assessed using the Nanodrop 1.0 or 2.0 spectrophotometer (Agilent).

### Real-time PCR analysis of enriched raw milk

Real-time PCR was performed on extracted DNA using 20 µl of the following mixture for each sample: PerfeCTa 1× qPCR ToughMix low ROX (QuantaBio), probes and primers at a final concentration of 0.3 µM (except for *ntb2* with a final concentration of 0.2 µM), completed with nuclease-free water. Either 5 µl DNA extracted using the InstaGene^TM^ protocol or 2 µl DNA extracted using the Lucigen protocol were added. Strain EDL933 (*stx1a*, s*tx2a*, *eae*) was used as positive control. Dsb or *ntb2* plasmid [33–36] was used as an inhibition control. The prepared samples were amplified using the CFX96 real-time detection system (BioRad) with the following program: 10 min at 95 °C (5 °C s^−1^); followed by 39 cycles of 15 s at 95 °C (2 °C s^−1^) and 60 s at 60 °C (2 °C s^−1^); and a final step of 30 s at 40 °C (5 °C s^−1^). Primers and probes are described in Table S2. All probes were labelled with 6-hexachlorofluorescein (HEX) or 6-carboxyfluorescein (FAM) and BHQ1 (black hole quencher) (Eurofins).

### Quantitative digital PCR (qdPCR) to quantify STEC O26 in enriched raw milk

qdPCR analysis was performed on the DNA extracted with the Lucigen protocol from enriched artificially contaminated raw milk samples. The Fluidigm BioMark system and the qdPCR 37 k IFC digital array microfluidic chips were used according to the manufacturer’s instructions (Fluidigm) and as previously described [37]. Reactions were performed in 6 µl per sample with 3 µl PerfeCTa 2× qPCR ToughMix low ROX (QuantaBio), 0.6 µl 20× GE sample loading reagent (Fluidigm), 0.3 µl 20× primer stock (containing 18 µM primers forward and reverse, and 4 µM probe), and 1.8 µl DNA, completed by nuclease-free water. A non-template control was included for each target. Real-time digital PCR was run using 6-carboxyfluorescein (FAM)- and black hole quencher (BHQ)-labelled probes. Amplification was performed with the following thermal profile: 2 min at 50 °C, 10 min at 95 °C, followed by 40 cycles of denaturation at 95 °C for 15 s and annealing at 60 °C for 60 s (rate of 2 °C s^−1^). Each fluorescent signal was acquired after the annealing step. Data were analysed using the Fluidigm digital PCR analysis software v4.1.2. The *wecA* genetic marker was used to quantify total *E. coli* and *wzx*_O26_ to quantify the artificially contaminated strain.

### MinION sequencing of raw milk

Libraries for MinION sequencing were prepared from the Lucigen DNA extracts using the LSK-SQK109 ligation kit and the EXP-NBD104 and/or EXP-NBD114 barcoding kits (Oxford Nanopore Technology) following the manufacturer’s recommendations, except that 2 µg genomic DNA was used as the starting material. Six to seven metagenomics samples were multiplexed and sequenced with a R.9.4.1 FLO-MIN106 flow cell (Oxford Nanopore Technology) on the Mk1B or Mk1C MinION sequencer (Oxford Nanopore Technology). Different amounts of pooled DNA libraries were loaded on each flow cell ranging from 96 to 530 ng.

### Sequencing data analysis

The sequencing runs were acquired without base-calling, and fast5 files output was selected. Raw fast5 files were base-called using guppy basecaller v4.4.2+ and demultiplexed using guppy barcoder v4.4.2+, with or without the –*require_barcodes_both_ends* option (Table S3).

A snakemake [38] pipeline, STECmetadetector (https://gitlab.com/bfr_bioinformatics/STECmetadetector), was developed in this study for user-friendly characterization of STEC strains from long-read metagenomics data. The main module of the STECmetadetector pipeline aims to assemble and characterize *E. coli* reads. First, barcodes and adapters are trimmed using porechop v0.2.4 (https://github.com/rrwick/Porechop). Short (< 1 kb) and low-quality reads (q-score <7) are filtered using NanoFilt v2.8.0 [39]. Nanoplot v1.39 is used to assess read quality from both raw and filtered reads [39]. Filtered reads are classified using the kraken2 classification tool v2.1.2 [40] and the Minikraken DB 8 GB (October 18 2017) database. The kraken2report file is converted into mpa format using krakentools v1.2 [40] (https://ccb.jhu.edu/software/krakentools/). Reads taxonomically assigned to *E. coli* are extracted using krakentools v1.2, mapped to detect *stx*, *eae* and O-group associated genes using minimap2 v.2.24 [41] and Center for Genomic Epidemiology (CGE) databases [42], and assembled with Flye assembler v2.9-b1768 [29]. The metagenome mode of Flye may be selected with the extra parameter *--meta*. The generated assembly is characterized using abricate v1.0.1 (https://github.com/tseemann/abricate) to detect serotype and the presence of virulence genes. Multilocus sequence type (MLST) is identified using mlst v2.19 (https://github.com/tseemann/mlst) and the *E. coli* 1 scheme [43]. The completeness, contamination and strain heterogeneity are screened using Checkm v1.1.3 [44]. Strainberry v1.1 [45] attempts to separate strains if multiple *E. coli* strains are suspected. A post-Strainberry module called Strainberry-parsing may be run to characterize the predicted *E. coli* strains. Several utility R scripts for parsing and summarizing results are integrated in the pipeline and use R packages mentioned in Table S4 [46].

To use the STECmetadetector pipeline, a sample sheet is required and can be provided by the user or created as mentioned in GitLab. STECmetadetector may be called by either using the Python *STECmetadetector.py* wrapper or by using the snakemake command line and editing the *config.yaml* file. The STECmetadetector pipeline can be also run on an HPC (high-performance computing) system with the *--cluster* option. All the pipeline’s software and database dependencies can be easily and reproducibly installed using conda [47]. Further information and detailed documentation can be found in the pipeline’s repository.

For the particular *eae*-positive STEC identification, *stx* and *eae* genes positions were extracted from the virulence file generated by abricate. Kraken2 output files (kraken2-classification.tsv and kraken2-output.tsv) from the pipeline were used to generate barplots and dotplots. For ease of presentation, only the most abundant genera have been included in the barplots. Flye assembly statistics were represented using dotplot graphs. All plots were generated using *ggplot2* v3.3.5 [48] and *reshape2* v1.4.4 [49] R packages on R v4.0.3 .

All versions of the software used here are reported in Table S4.

### Statistical analysis

The Kruskal–Wallis non-parametric test and its post hoc Mann–Whitney test were used for statistical analysis, with alpha error being fixed at 5 %. The resulting *P* values were represented on an R-generated boxplot using the *ggpubr* R package v0.4.0 (https://rpkgs.datanovia.com/ggpubr/) and the --*stat_compare_means* function on R v4.0.3 and R Studio v1.3.1093.

## Results

### Identification of the minimum coverage required to identify *eae*-positive STEC using MinION sequencing

**Fig. 1.**
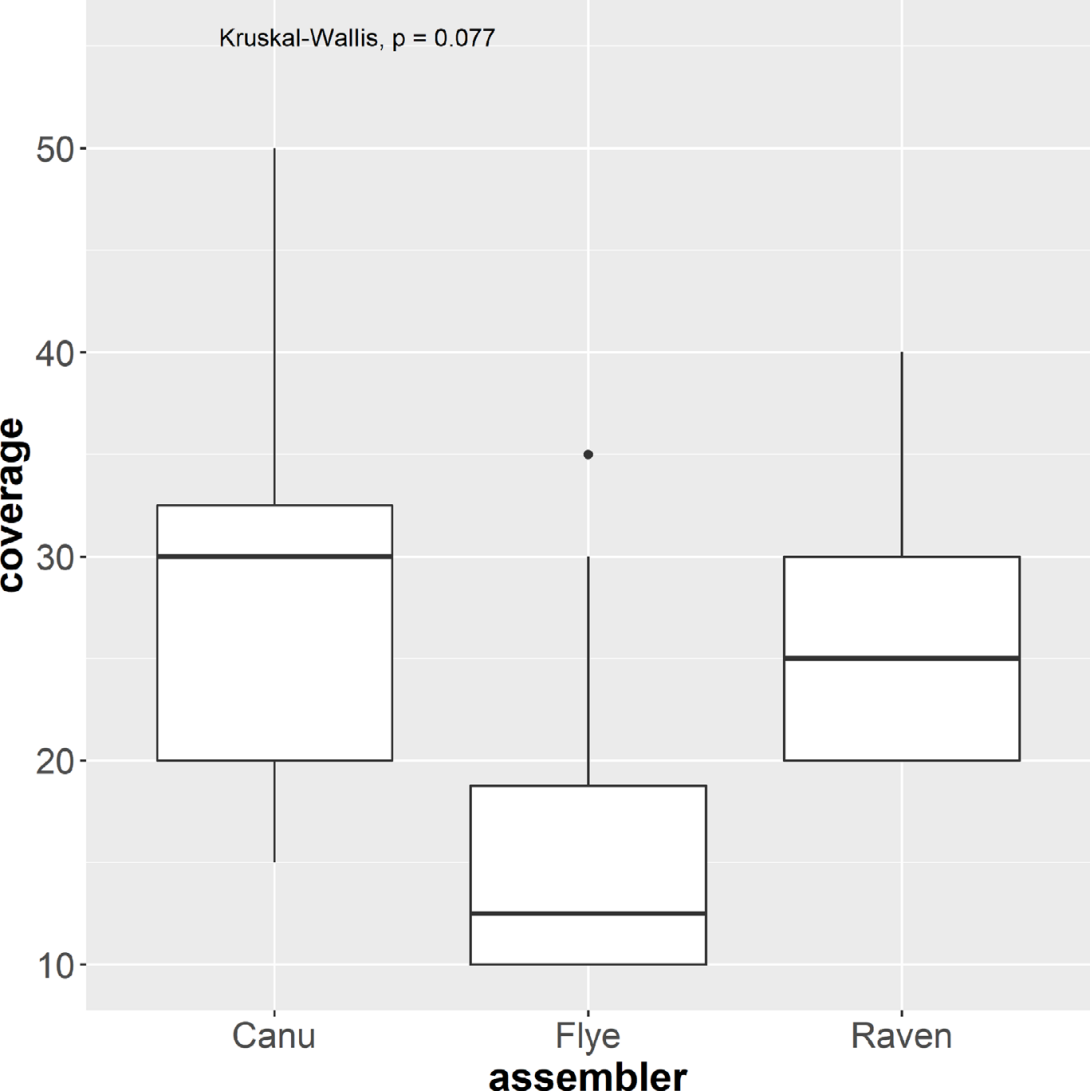
Boxplot representing the minimal coverage required to assemble *eae*-positive STEC. The minimal coverage at which *stx* and *eae* were co-localized on the same contig is represented depending on the long-read assembler used on subsampled MinION data of 10 *eae*-positive STEC strains. The minimal coverage required for *eae*-positive STEC identification was confirmed when *stx* and *eae* genes were found on the same contig in all of the following assemblies with higher coverages (one exception was accepted). Flye was the most efficient to assemble *eae*-positive STEC with a genome coverage up to 35×, although the difference with Canu and Raven assemblers was not significant (Kruskal–Wallis test, *P* value >0.05).

We first aimed to determine the minimum coverage required to assemble the *stx* and *eae* genes on the same contig. For this purpose, we generated *in silico* assemblies using sub-sampled MinION datasets sequenced from *eae*-positive STEC isolates (*n*=10) using three different long-read assemblers. While the difference between the assemblers’ performance was not significant (Kruskal–Wallis, *P*=0.077), Flye performed best in identifying *eae*-positive STEC strains from a genome coverage of 10× to 35× in eight strains (Fig. 1). Canu assembled seven strains with genome coverage varying from 15× to 50× and, lastly, Raven required a minimum coverage of 20× for *eae*-positive STEC strain identification, but did not assemble *stx* and *eae* genes on the same contig for five strains (Fig. 1). For one strain, all assemblers failed to assemble both virulence genes on a single contig, even at a high coverage. Based on the results, the minimum coverage to ensure *eae*-positive STEC identification from genome assembly for most strains was 35× with Flye assembler, in the default mode.

### Generation of a snakemake pipeline for STEC characterization from long-read metagenomics data

#### STECmetadetector pipeline

**Fig. 2.**
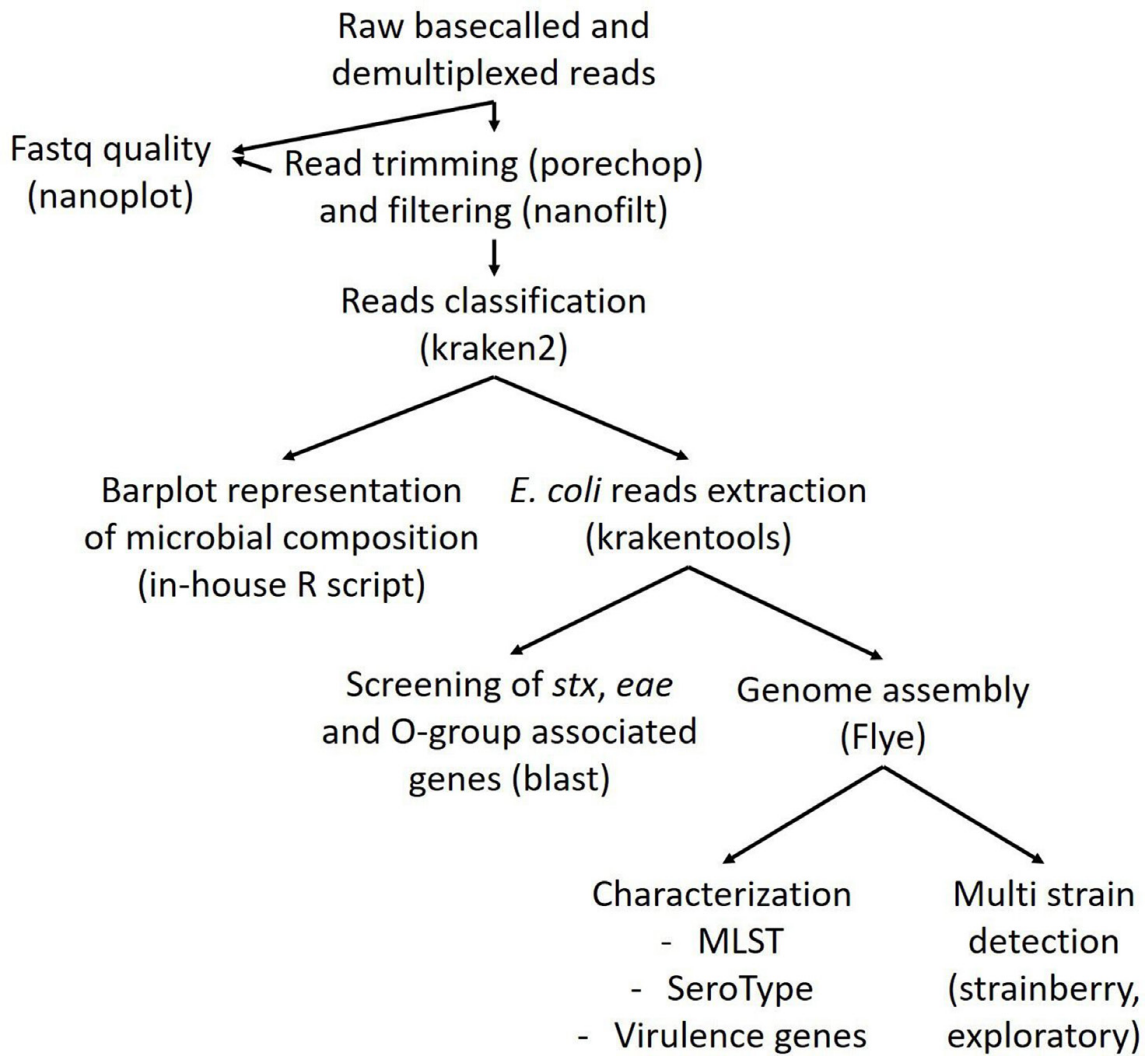
Presentation of the different steps performed by the STECmetadetector pipeline for *eae*-positive STEC identification from long-read metagenomics data.

We generated a complete and open-source snakemake pipeline (https://gitlab.com/bfr_bioinformatics/STECmetadetector), which orchestrates appropriate state-of-the-art tools to consistently and reproducibly characterize STEC strains from metagenomics data using an assembly-based approach (Fig. 2) [38]. All relevant information from the different modules is aggregated into a complete HTML report file. All steps of this pipeline are presented in Methods (in the Sequencing data analysis section) and represented in Fig. 2.

#### Pipeline results on real metagenomics data

The STECmetadetector pipeline was used to select pertinent data for STEC characterization in artificially contaminated raw milk samples. Short and low-quality reads (length <1 kb and q-score <7) were filtered, which resulted into the retention of 8.49–57.5 % of all reads but in more than 42.73 % of the generated data (in bases) (mean=73.85±11.88 %, *n*=31). Most filtered reads were taxonomically assigned (88.73–99.77 %, *n*=30), except for one sample (Milk5_37+A) with a very low DNA concentration extracted (below limit of quantification; classified reads=51.55 %, *n*=1). Between 0.03 and 46.24 % and between 13.9 and 80.62 % of classified reads were assigned as *E. coli* in uncontaminated and artificially contaminated raw milk samples, respectively (mean=5.73 and 64.46 %, ±14.33 and 18.1 %, respectively; *n*=10+21). Flye assembly on *E. coli* extracted reads from artificially contaminated samples showed a mean total assembly length of 5.92 Mb (4.96–6.4±0.24 Mb, *n*=21). The *wzx*_O26_, *stx* and *eae* genes were co-localized on the same contig in 19 Flye assemblies (*n*=21; Table S3).

### Determination of the optimal enrichment conditions for multiplying *eae*-positive STEC in raw cow's milk

Based on *in silico* results presented in Results (in the section Identification of the minimum coverage required to identify *eae*-positive STEC using MinION sequencing), we established that an enrichment step was necessary to achieve the minimal coverage required for STEC assembly. We then determined the best enrichment conditions for *eae*-positive STEC multiplication and identification in raw milk using long-read metagenomics. STEC-negative raw milk was artificially contaminated with 0.5×10^3^ c.f.u. ml^−1^ (Milk1) and 0.5×10^2^ c.f.u. ml^−1^ (Milk2 and Milk3) of one STEC strain per ml of raw milk. Four different conditions were compared with samples being incubated at either 37 or 41.5 °C, with or without acriflavine supplementation (final concentration of 12 mg l^−1^). Acriflavine is an antibiotic targeting Gram-positive bacteria recommended for STEC enrichment in milk and dairy products by the ISO/TS 13136 : 2012.

**Fig. 3.**
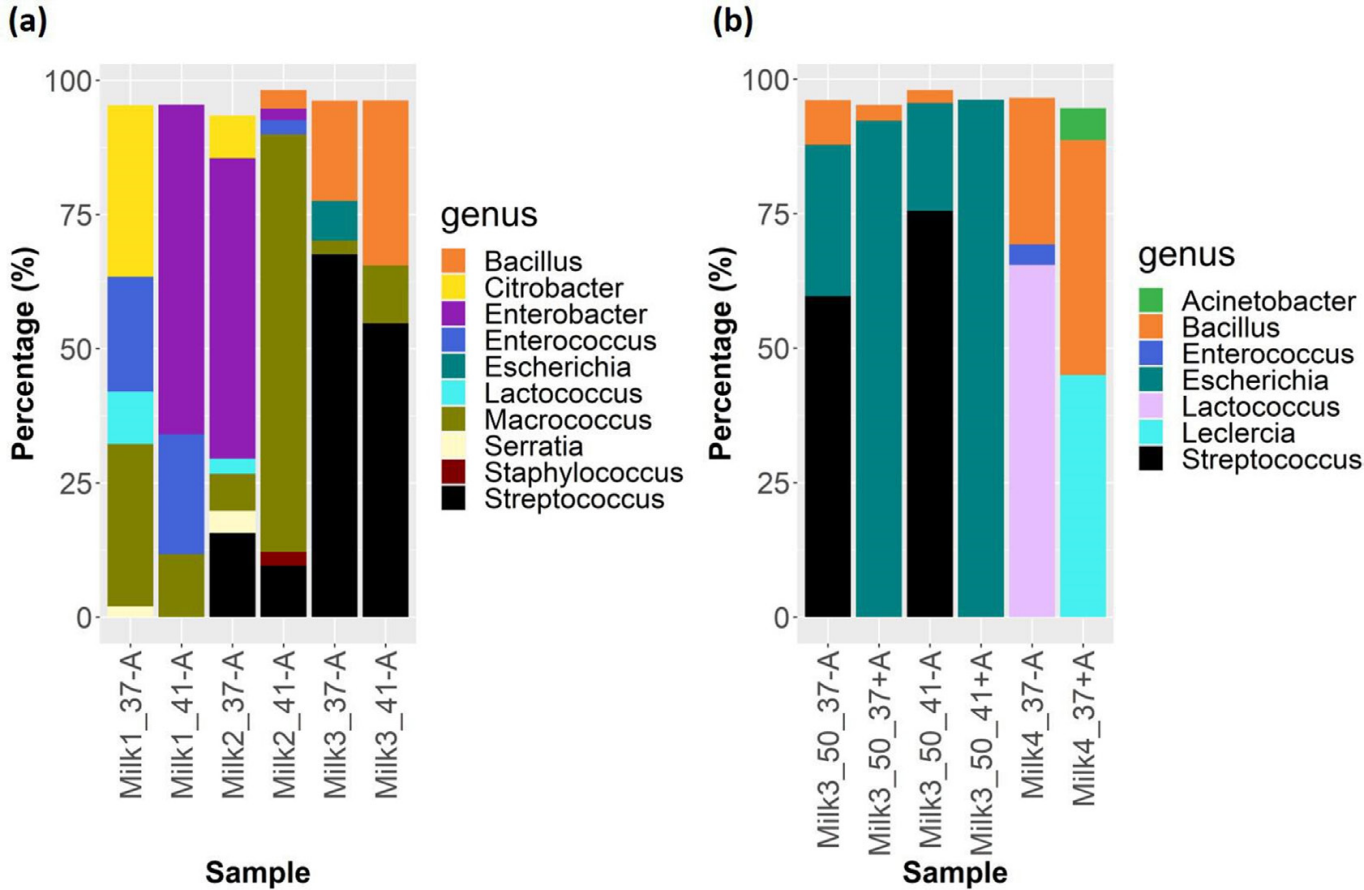
Barplots representing the enrichment temperature and acriflavine supplementation impact on the background flora. MinION-sequencing data were analysed using the STECmetadetector pipeline. (a) Barplot representing the most abundant genera (>2 %) in three natural raw milk samples enriched at 37 °C (_37) or at 41.5 °C (_41) without acriflavine (-A) supplementation. (b) Barplot representing the most abundant genera (>2 %) in two enriched milk samples. One was inoculated at the concentration of 0.5×10^2^ c.f.u. STEC strain ml^−1^ in raw milk (Milk3_50) and enriched at 37 °C (_37) or 41.5 °C (_41) with (+A) or without (-A) acriflavine supplementation. The second milk sample was enriched at 37 °C with (Milk4_37+A) or without (Milk4_37-A) acriflavine supplementation.

While the enrichment temperature had no impact on the inoculated strain growth (Table 2), we observed a changing composition of the background flora in non-contaminated raw milk samples, yet without a clear pattern (Fig. 3a). Similarly, acriflavine did not negatively affect the growth of both 4712-O26 and 6423-O26 *eae*-positive STEC strains (Table 2). However, it strictly reduced the proportion of Gram-positive bacteria such as *Lactococcus* spp. (Milk4) and *Streptococcus* spp. (Milk3) that were dominant in those samples, from 65.59 and 59.68 % to less than 1 and 2 % of the filtered reads, respectively. By preventing Gram-positive bacterial growth, the growth of Gram-negative bacteria was favoured and particularly that of the inoculated STEC strain (Fig. 3b). One notable example was in an artificially contaminated raw milk containing a naturally high proportion of *Streptococcus* spp., which was reduced from 59.68 % to less than 2 % after acriflavine supplementation, while we observed a drastic increase of *E. coli* reads detected from less than 24.76 % to more than 87.57 %, at both 37 and 41.5 °C (Fig. 3b). However, the impact of acriflavine on *Bacillus* was different depending on the milk samples (Fig. 3b). Overall, these results clearly showed that acriflavine supplementation may improve the sensitivity of the method.

**Table 2.** Concentration of STEC O26 in raw milk (c.f.u. ml^−1^) and quantification in raw milk enriched in four different conditions by qdPCR (copies ml^−1^)

Concentration of STEC O26 in raw milk before enrichment (determined by plating on agar plates) [c.f.u. ml^−1^]	Enrichment conditions	Quantification of STEC O26 in enriched milk by qdPCR [copies ml^−1^]
1.06×10^2^ – 0.625×10^3^ (*n*=3)	37 °C with acriflavine	2.59×10^8^ – 1.45×10^9^
1.00×10^2^ – 0.72×10^3^ (*n*=3)	37 °C without acriflavine	6.77×10^8^ – 1.24×10^9^
1.06×10^2^ – 0.625×10^3^ (*n*=3)	41.5 °C with acriflavine	4.47x10^8^ – 6.82×10^8^
1.00×10^2^ – 0.72×10^3^ (*n*=3)	41.5 °C without acriflavine	6.77×10^8^ – 9.95.10^8^

### Determination of the identification limit for an *eae*-positive STEC strain in enriched raw milk using long-read metagenomics

**Fig. 4.**
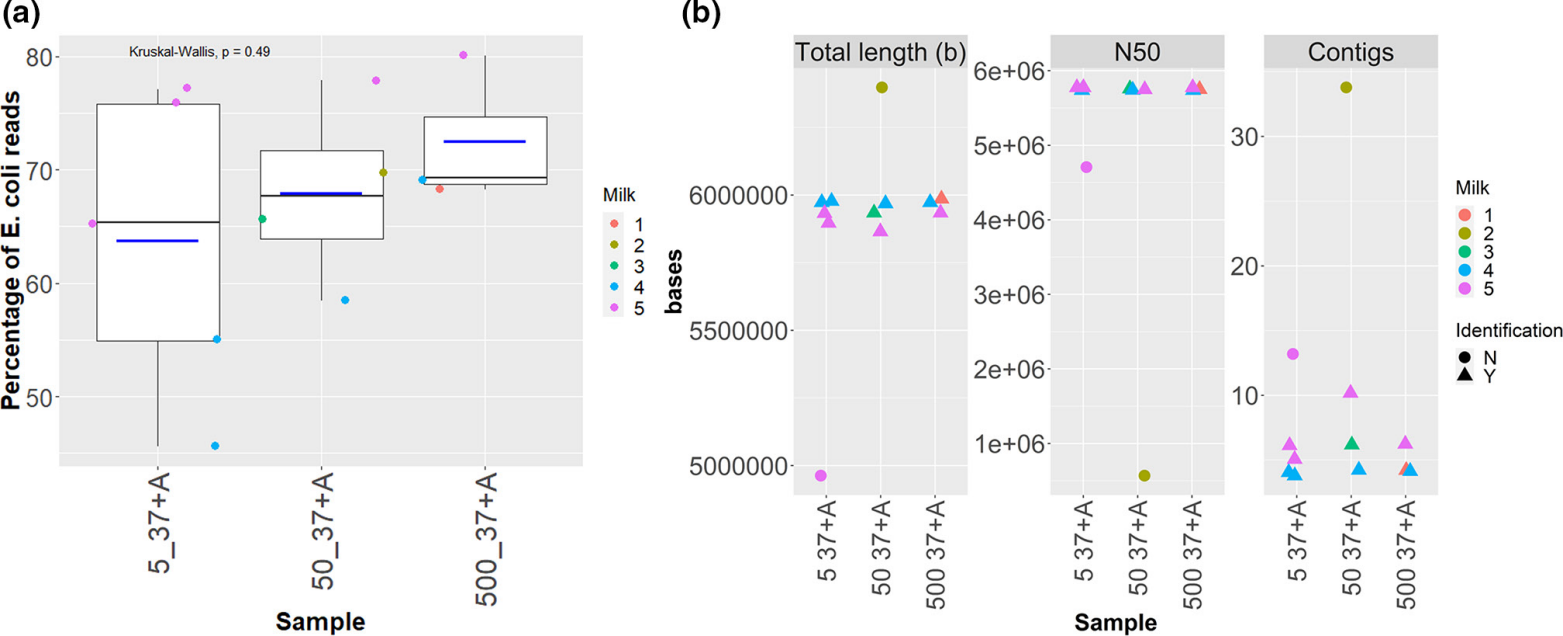
*
E. coli* reads proportion and *E. coli* assembly metrics obtained from artificially contaminated raw milk. Raw milk samples were artificially contaminated with STEC at the concentrations of 0.5×10^3^ c.f.u. ml^−1^ (*n*=3; 500), 0.5×10^2^ c.f.u. ml^−1^ (*n*=4, 50) or 0.5×10^1^ c.f.u. ml^−1^ (*n*=5, 5) of raw milk and enriched at 37 °C in the presence of acriflavine (37+A). (a) Boxplot representing the percentage of extracted *E. coli* reads. (b) Dotplot representing assembly metrics obtained from Flye assemblies on extracted *E. coli* reads. A triangle represents the identification of *stx* and *eae* genes on the same contig, whereas their identification on two different contigs is represented using a circle. Milk batches are represented using different colours.

Using the selected enrichment conditions, STEC-negative raw milk was artificially contaminated with one STEC strain at concentrations from 0.5×10^1^ to 0.5×10^3^ c.f.u. ml^−1^ in raw milk and enriched for 18–20 h. Table 3 shows the contamination levels of STEC O26 (c.f.u. ml^−1^ in milk) as estimated by optical density and calculated by plating on agar plates. Table 3 also reports quantification of the total *E. coli* (*wecA*) and the spiked STEC O26 (*wzx*_O26_) in enriched milk by qdPCR (copies ml^−1^ in enriched milk). DNA extracted from enriched milk samples was sequenced and processed with the STECmetadetector pipeline. The results showed, as expected, that the mean proportion of *E. coli* reads increased with the STEC strain concentration in raw milk, with a percentage of *E. coli* reads of 63.75 % (±13.56, *n*=5), 67.93 % (±8.1, *n*=4) and 72.53 % (±6.54, *n*=3) of filtered reads, for initial STEC concentrations of 0.5×10^1^, 0.5×10^2^ and 0.5×10^3^ c.f.u. ml^−1^ in raw milk, respectively (Fig. 4a).

**Table 3.** Concentration of STEC O26 in raw milk before and after enrichment Quantification of *wecA* and *wzx*_O26_ genetic markers in enriched raw milk (37 °C with 12 mg acriflavine l^−1^) was determined by qdPCR (copies ml^−1^ in enriched milk). qdPCR results for each non-contaminated raw milk sample enriched at 37 °C without acriflavine are also presented.

Calculated concentration of STEC O26 inoculum (determined by plating on agar plates) (c.f.u. ml^−1^)	Estimated concentration of STEC O26 in raw milk before enrichment (determined by OD_600_) (c.f.u. ml^−1^)	Quantification of total *E. coli* (*wecA*) in enriched milk by qdPCR (copies ml^−1^)	Quantification of STEC O26 (*wzx*_O26_) in enriched milk by qdPCR (copies ml^−1^)
1.25x10^3^ – 1.78×10^3^	500 (*n*=4)	8.65×10^8^ – 2.37×10^10^	1.16×10^9^ – 2.62×10^10^
1.67x10^2^ – 2.63×10^2^	50 (*n*=5)	4.94×10^8^ – 5.13×10^9^	2.59×10^8^ – 2.92×10^9^
1.33×10^1^	5 (*n*=8)	8.59×10^8^ – 1.92×10^10^	2.41×10^7^ – 1.04×10^10^
0	0 (*n*=5)	Without acriflavine: 0–1.37×10^9^	Without acriflavine: 0

Fig. 4b represents assembly metrics from artificially contaminated samples enriched at 37 °C with acriflavine supplementation. In 10 assemblies (*n*=12 samples), the serotype-associated genes and virulence genes *stx* and *eae* were detected on the same contig, with *stx* and *eae* being at a distance of 1.9 Mb. In two samples, the *eae*-positive O26 STEC was not identified. One sample (Milk2_50_37+A) showed a fragmented assembly with 34 contigs and a high genome coverage (111×). When we sub-sampled *E. coli* reads to lower genome coverage, still *stx* and *eae* could not be assembled on the same contig, although the filtered reads N50 value (13 136 bp) and the quality (12.6) seemed acceptable. The pathotypes predicted by the STECmetadetector pipeline was ‘EHEC?’ since *stx* and *eae* genes were detected but could not be assembled on the same contig. In the second sample (Milk5_5_37+A_1), a commensal *E. coli* genome was assembled instead of the STEC that was inoculated at a low contamination level of 5 c.f.u. (ml raw milk)^−1^ (Fig. 4b).

Our results showed that the identification of the inoculated STEC strain was possible from an STEC concentration as low as 5 c.f.u. (ml raw milk)^−1^ (*n*=4/5) [corresponding to 2.59×10^8^ – 2.62×10^10^ copies (ml enriched milk)^−1^] (Table 3) under the above-mentioned enrichment conditions Although commensal *E. coli* were naturally present and quantified to 1.37×10^9^ copies ml^−1^ post-enrichment in non-artificially contaminated milk (Milk5; Table 3), the inoculated STEC O26 strain growth [contamination level of 5 c.f.u. (ml raw milk)^−1^] was sufficient to fully characterized its genome in 2/3 replicates. In the third replicate (Milk5_5_37+A_1), a commensal *E. coli* strain grew better than the STEC and was fully assembled perturbing STEC strain characterization using assembly (*n*=1/3) (Fig. 4b).

### Long-read metagenomics revealed that raw milk microbiota impacts the growth of *eae*-positive STEC

**Fig. 5.**
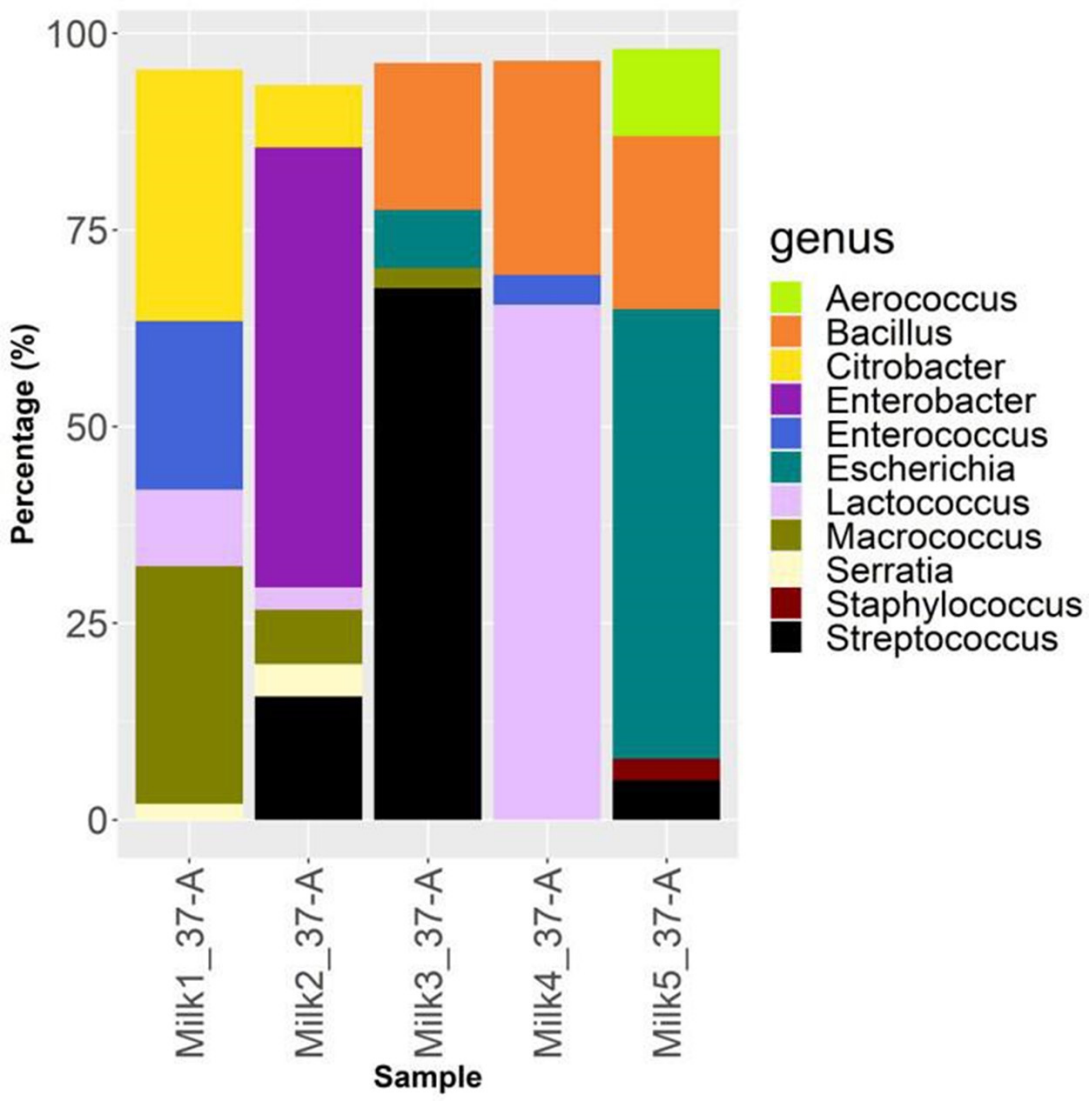
Barplot representing the most abundant bacterial species (>2%) in all studied raw cow's milk samples after enrichment at 37 °C without acriflavine supplementation (37-A, *n*=5). MinION data obtained from five natural raw milk samples were analysed with the STECmetadetector pipeline and the composition presented.

In order to perform all the artificial contamination experiments on fresh raw milk, five different fresh raw milk samples were used in this study. Three milk samples (1, 2 and 3) were bought from a commercial farm in France and two milk samples (4 and 5) were collected from an experimental farm in Germany. Long-read metagenomics revealed different raw milk microbiota for all investigated enriched milk samples. *E. coli* reads were detected only in non-contaminated raw milk samples Milk3 and Milk5 representing 6.8 and 46.23 % of filtered reads, respectively (Fig. 5). The use of acriflavine affected the survival of certain Gram-positive bacteria, which consequently increased the proportion of STEC. One pertinent example is Milk3 in which *Streptococcus* spp. dominated and limited the STEC growth in the absence of acriflavine (Fig. 3b). However, acriflavine did not completely prevent the growth of Gram-positive bacteria, as observed with Milk4 that contained a high proportion of *Bacillus* spp. (Fig. 3b). While in most samples the STEC strain growth was not negatively affected in acriflavine-supplemented enrichments, in artificially contaminated milk sample 4 the observed percentage of *E. coli* reads post-enrichment was lower than expected (Fig. 4a).

Commensal *E. coli* strains present in raw milk did not appear to compromise the growth of the STEC O26 inoculum. Indeed, two non-inoculated milk samples (Milk3_37 A and Milk5_37 A) had detectable levels of *E. coli* (1.97×10^7^ and 1.37×10^9^ copies ml^−1^ total *E. coli *, respectively) post-enrichment at 37 °C without acriflavine using qdPCR. Characterization of *E. coli* reads from milk samples Milk3_37 A and Milk5_37 A revealed the presence of commensal strains of serotype O149:H20 in Milk3_37 A, and both O185:H2 [sequence type (ST)2522] and O8:H19 (ST162) strains in Milk5_37 A enriched at 37 °C without acriflavine (Table S3). The natural presence of O185 and O8 strains in raw milk 5 was confirmed by qdPCR analysis (8.75×10^8^ and 1.84×10^7^–3×10^7^ copies ml^−1^, respectively).

In artificially STEC O26 contaminated milk batch 3, *wzx*_O149_ was not detected when enriched at 37 °C in the presence of acriflavine, but it was detected (7.43×10^5^ copies ml^−1^) when enriched at 37 °C in the absence of acriflavine. Commensal strains markers O185 and O8 were not detected nor assembled in artificially contaminated raw milk samples from milk batch 5, except in one of the three biological replicates of Milk5 inoculated at 0.5×10^1^ c.f.u. ml^−1^. The O185 marker was quantified to 4.55–8.84×10^9^ copies ml^−1^ by qdPCR. Additionally, this commensal strain (O185:H2) was assembled, while the inoculated STEC was not. Although read mapping detected O26 as the major O-group in all other artificially contaminated samples, in this sample (Milk5_5_37+A_1) more reads mapped to O185 (154×) than O26 (13×) (Table S3). Analysis on this replicate showed a difference of approximately one log (copies ml^−1^) between total *E. coli* and the inoculated strain with both real-time PCR (i.e. a difference of 3 *C*_t_ values for both *stx* and *eae* – specific to the inoculated strain – and *wecA* – common to both natural and inoculated strains) and qdPCR [estimated between 2.41×10^7^ and 1.09×10^8^ copies ml^−1^ for O26 marker specific of the inoculated strain, and between 8.59×10^8^ and 1.92×10^9^ copies ml^−1^ for *wecA* (Table 3)]. Similarly, in this replicate in which the O185:H2 strain was assembled, qdPCR results confirmed a higher level of the O185 strain [6.52×10^8^–10.09×10^10^ copies ml^−1^ of O185 (*wzx*_O185_)] compared to the STEC O26 strain. The presence of commensal *E. coli* did not affect the growth of the STEC O26 strain in 2/3 replicates of raw milk artificially contaminated with 5 c.f.u. ml^−1^ and enriched in acriflavine-supplemented BPW at 37 °C. However, it hindered its identification using an assembly-based approach in one replicate.

## Discussion

Food-borne pathogens are a worldwide concern. Recently, different approaches for identification of food-borne pathogens were developed. While metagenomics is widely used for the detection and characterization of food-borne pathogens like *Salmonella* or *Listeria *, its efficacy to distinguish STEC that may be *eae*-positive or -negative in complex samples like raw milk is questionable [17]. *E. coli* is a member of the cow gut microbiota, in which commensal and/or pathogenic strains of different pathotypes may coexist. Cookson and colleagues detected as many as 50 different *E. coli* strains in a single sample of cattle faeces [50]. STEC harbour various virulence markers, like the *eae* gene, that are detectable by real-time PCR (qPCR) in complex samples like raw milk [34]. However, it is not resolvable whether these genes belong to the same strain or are present in different strains, and isolation is required to confirm the presence of all virulence factors in a single strain (strain characterization). STEC isolation is laborious, time-consuming and frequently unsuccessful in raw milk, because of the background flora and a lack of a STEC-specific isolation medium. The diversity of STEC emphasizes the need for STEC characterization methods that circumvent the isolation problems posed by current methods. The aim of this study was to explore metagenomics as an innovative STEC isolation-independent method able to identify and characterize STEC in enriched raw milk that could be applied to characterize *stx*-positive samples as an alternative to strain isolation.

So far, only a few studies that describe the detection and characterization of STEC using long-read metagenomics are available [18, 19]. Only one of them explored metagenomics in beef for specifically identifying *eae*-positive STEC of serotype O157:H7 that carry both *stx* and *eae* genes [18]. Given that these genetic markers, *stx* and *eae*, can also be carried by non-pathogenic *E. coli* strains or other bacterial species, this objective represents a real challenge. In this study, we developed a straightforward strategy different from that described by Buytaers *et al.* to identify *eae*-positive STEC using long-read metagenomics of artificially contaminated raw milk [18]. While they used a DNA walking approach linking *stx* and *eae* virulence genes to an *Escherichia* genome, we adopted an assembly-based method. This approach could be applied to characterize any type of *E. coli* contaminating a food matrix, regardless of the existence of reference strains for this pathotype. To mimic real samples, two *eae*-positive STEC O26 isolated from raw cow's milk were selected for this study. Indeed, O26 is one of the STEC serogroups most frequently found in clinical cases in Europe, and is also highly dominant in French and German raw milk [9, 34]. Firstly, we developed a wet-lab method to prepare the samples for long-read sequencing. Secondly, we determined the sensitivity of the method for identification of *eae*-positive STEC after enrichment of artificially contaminated raw milk using long-read metagenomics. Thirdly, we developed a complete and automated analysis pipeline for STEC strain characterization from MinION sequencing data.

Previous studies have determined that the detection limit of STEC strains using MinION sequencing was around 10^7^ c.f.u. ml^−1^ in wastewater [19]. These contamination levels are generally not reached in naturally contaminated raw milk samples without incubating the sample to get *E. coli* growing to a detectable level. Therefore, an enrichment step appears necessary in order to identify *eae*-positive STEC strains from dairy products. As demonstrated in this study, Flye performed best in assembling *eae*-positive STEC (*n*=10), although below a coverage of 35×; *stx* and *eae* virulence genes were not consistently co-localized on a single contig; thus, providing a potential misidentification. A STEC genome at a coverage of 35× represents around 192.5 Mb (genome size=5.5 Mb). Shotgun metagenomics studies on raw milk without enrichment revealed tremendous proportions of host DNA varying from 82.1 to 99.5 % of sequenced reads [51–53]. Consequently, around 2 % of the total DNA sequenced from raw milk in these conditions would correspond to bacterial DNA, which represents 60 Mb of an estimated 3 Gb output sequencing run. By supposing the presence of *E. coli* to be 100 % of the bacterial DNA in the milk sample, 60 Mb would represent 10× STEC genome coverage, which is clearly not sufficient for a reliable STEC genome assembly and characterization. In addition, long reads are error-prone, and need to be filtered on quality and length, which, along with the background flora, contributes to reduce the amount of pertinent data available for analysis. Commercial host DNA-removal kits allow a significant increase in the ratio of bacterial DNA reads compared to host DNA reads [51]. However, removing 98 % of the extracted DNA can lead to technical difficulties related to the amount of DNA necessary for MinION sequencing (1–2 µg genomic DNA versus only 1 ng genomic DNA for Illumina sequencing). Consequently, an incubation step to get viable *E. coli* growing to a detectable level was preferred, because it not only considerably reduces the proportion of host DNA while keeping viable STEC cells, but also enables sample multiplexing on a flow cell, which dramatically reduces the cost of long-read metagenomics.

Comparison of four different enrichment conditions revealed that even though no difference in terms of *eae*-positive STEC strain identification was observed, the use of acriflavine as recommended by the ISO/TS 13136 : 2012 [11] was relevant for enrichment efficacy, especially in raw milk containing Gram-positive bacteria. Although the precise benefit of acriflavine during enrichment is still debated [54, 55], in this study, acriflavine was used during the enrichment process because it did not appear to negatively influence the growth of the inoculated strains, while preventing the growth of some of the background flora. Further studies on the impact of acriflavine on the growth of STEC strains from various serotypes would be nonetheless required. We tested two different enrichment temperatures recommended for non-O157 and O157 STEC strains respectively, 37 and 41.5 °C. The inoculated O26:H11 STEC strains were not affected by either 37 or 41.5°C. In 2008, Baylis demonstrated that the temperature of enrichment alone does not affect the growth of STEC strains, even though it may help reduce the background flora [56]. Here, acriflavine supplementation was more effective at reducing the Gram-positive background flora than performing the enrichment step at 41.5 °C. Hence, all the samples further analysed were enriched at 37 °C for 18–20 h in the presence of acriflavine. Although it was not tested here, it might be theoretically possible to reduce the enrichment time while maintaining sufficient levels of cells for *eae*-positive STEC characterization using long-read metagenomics.

In this study, we aimed to test the long-read metagenomics potential on STEC assembly from metagenomics samples for strain characterization. Under the optimized enrichment conditions, STEC-negative raw milk samples artificially contaminated with an O26 *eae*-positive STEC strain, at initial concentrations from 0.5×10^1^ to 0.5×10^3^ c.f.u. ml^−1^ in raw milk, could be clearly identified by MinION long-read metagenomics. The percentage of reads assigned to *E. coli *, as expected, increased with STEC concentration. It is noteworthy that the presence of certain bacterial genera, even in the presence of acriflavine, may adversely affect the growth of STEC strains (i.e. Milk4, which contained 27.25 % of *Bacillus *, showed lower percentages of *E. coli* reads after artificial contamination using three concentrations of 4712-O26 culture in raw milk and enrichment with acriflavine).

For easy data analysis of long-read metagenomics, we developed a strain-level characterization pipeline specifically for STEC. The pipeline aims at identifying in particular STEC containing *stx* and *eae* genes in the same strain. This pipeline gathers appropriate tools for STEC genome characterization and enables reproducible analysis on different metagenomics runs. Basecalling and demultiplexing are currently not part of the STECmetadetector pipeline, but should be run on a system enabling Graphics Processing Units (GPUs) to considerably reduce basecalling time. We recommend doing precise basecalling using the high-accuracy or super high-accuracy model, which may help reduce the error-rate and better characterize the strain. Nevertheless, direct basecalling, using the fast basecalling model, and demultiplexing, may be performed on-board using either the MinIT in combination with Mk1B or the Mk1C device, if no GPUs are installed. Once the basecalling and demultiplexing are done, the optimized pipeline designed in this study is useful to fully and precisely characterize *E. coli* strains. Extraction of *E. coli* reads before assembly significantly reduces the overall amount of data and optimizes resource consumption. Although the STECmetadetector pipeline was designed for identifying *eae*-positive STEC, it may be used to characterize other *E. coli* pathogroups and may prove particularly useful for characterization of hybrid or heteropathotype strains.

Using the STECmetadetector pipeline designed in this study, we were able to identify *eae*-positive STEC from long-read metagenomics data and obtained the complete chromosome in one contig from 2.59×10^8^ copies (ml enriched raw milk)^−1^. Flye assembler, included in the pipeline, not only was shown to be one of the best for *E. coli* genome assembly, but also is currently the only long-read assembler enabling metagenome assembly (metaFlye) and offering the best balance regarding ‘time/results quality’ [25, 57]. The metaFlye assembler was released to assemble similar reads belonging to different bacterial species present in metagenomics samples but is not designed to distinguish different strains from the same species. To reduce analysis complexity from metagenomes, in part caused by the presence of other bacterial species and potentially multiple *E. coli* strains, we filtered and assembled *E. coli* reads only. In line with Vicedomini and colleagues [45], we showed that even in the presence of several *E. coli* strains in the same sample, Flye usually assembled only one *E. coli* genome completely. This is one major drawback of current assemblers, which may hinder the development of this method. The presence of additional small contigs of different coverages, as well as multiple O-groups and/or multiple alleles of the different MLST genes, may indicate the presence of different *E. coli* strains in the sample.

It is probable that the presence of other *E. coli* strains may compromise *eae*-positive STEC identification since the *eae* and *stx* genes may be distantly located on the *E. coli* chromosome (i.e. 1.9 Mb here), and assemblers are currently not systematically able to distinguish strains. Although we did not aim to analyse the impact of commensal *E. coli* strains (*stx*- and *eae*-) on the STEC strain growth, we had to use different milk samples to perform all the artificial contamination experiments on fresh milk in which commensal *E. coli* were present in 2/5 milk samples. We noticed that it affected the STEC assembly in 1/3 replicates inoculated with 5 c.f.u. (ml raw milk)^−1^ (Milk5_5_37+A_1). The inoculation level of 5 c.f.u. ml^−1^ thus most likely represents the limit of STEC characterization by long-read metagenomics in the presence of commensal *E. coli.* A recent strain-separation tool named Strainberry was used to distinguish multiple *E. coli* strains [45]. If Strainberry predicted the presence of multiple strains, the post-Strainberry module was used to characterize each resulting strain. We were able to separate two different commensal *E. coli* genomes in uncontaminated raw milk using Strainberry. Identifying a STEC strain in the presence of commensal *E. coli* strains at an initial low inoculation level of 5 c.f.u. ml^−1^ in raw milk was successful in two out of three replicates using the optimized assembly-based method described in our study. The outlier was only observed for the replicate where the inoculated strain growth was limited compared to the two other replicates, as confirmed by qdPCR and real-time PCR analysis. It is probable that these conditions constitute the lower limit and that, for this replicate, slightly less than 5 c.f.u. ml^−1^ were sampled and inoculated, allowing a better growth of the O185 strain.

The presence of commensal *E. coli* strains together with STEC are not the only challenge for STEC growth in complex matrices. Raw milk microbiota is very variable because of the milk’s high content of nutrients and the diverse sources of contamination from the environment within the production chain leading to different bacterial colonization [58, 59]. Gram-positive bacteria, for example *Lactococcus lactis* and *Streptococcus* spp., generally dominate in milk microbiota. A high proportion of *Lactococcus* spp. reads was detected in one sample (Milk4_37 A) and acriflavine prevented its growth during enrichment. Similarly, in another milk sample (Milk3_37 A) many reads were assigned to *Streptococcus uberis *, which is known to be a cause of mastitis and may acidify the medium, but they were not detected in an acriflavine-supplemented enrichment sample [60]. Despite certain variability in the raw milk microbiota, long-read metagenomics seems to be a promising approach for identification of pathogenic STEC in enriched raw milk.

In conclusion, the use of long-read metagenomics for food-borne pathogen identification is emerging and constantly evolving. The work of Buytaers and co-workers was a proof-of principle for STEC identification using long-read metagenomics in beef samples [18]. By using a different approach, we were able to reveal the co-localization of O26:H11 serotype-associated genes, *stx* and *eae*, in the same strain with the objective to identify *eae*-positive STEC strains from artificially contaminated raw milk samples. The complete approach with optimized enrichment conditions for the growth of *eae*-positive STEC strains, and its characterization using long-reads metagenomics using an adequate pipeline (STECmetadetector), is a significant improvement for STEC identification in metagenomes. The methodology has potential to be applied for characterizing STEC directly in food samples. However, its widespread application in monitoring will be possible only when new, more strain-aware assemblers become available.

## Supplementary Data

Supplementary material 1

Supplementary material 2

Supplementary material 3

Supplementary material 4
